# Immuno-Golgi as a Tool for Analyzing Neuronal 3D-Dendritic Structure in Phenotypically Characterized Neurons

**DOI:** 10.1371/journal.pone.0033114

**Published:** 2012-03-12

**Authors:** Luísa Pinto, António Mateus-Pinheiro, Mónica Morais, João Miguel Bessa, Nuno Sousa

**Affiliations:** 1 School of Health Sciences, Life and Health Sciences Research Institute, University of Minho, Braga, Portugal; 2 PT Government Associate Laboratory, Life and Health Sciences Research Institute/3B's, Braga/Guimarães, Portugal; University of Nebraska Medical Center, United States of America

## Abstract

Characterization of neuronal dendritic structure in combination with the determination of specific neuronal phenotype or temporal generation is a challenging task. Here we present a novel method that combines bromodioxyuridine (BrdU) immunohistochemistry with Golgi-impregnation technique; with this simple non-invasive method, we are able to determine the tridimensional structure of dendritic arborization and spine shape of neurons born at a specific time in the hippocampus of adult animals. This analysis is relevant in physiological and pathological conditions in which altered neurogenesis is implicated, such as aging or emotional disorders.

## Introduction

The unique phenomenon of adult neurogenesis adds a new dimension to neuroplasticity in the adult brain [1], [2]. By definition, adult neurogenesis comprises proliferation, migration and differentiation phases, before the newly-born neurons are incorporated in pre-existent neuronal networks. Using distinct tools to monitorize adult neurogenesis many researchers try to unveil the functional implications of adult neurogenesis. Strikingly, this neuroplastic phenomenon is disrupted in many disorders [3], [4] and, thus, its analysis, particularly when combined with complementary methods became of great relevance to dissect the underlying mechanisms of those disorders.

Neuronal proliferation and survival can be regulated by several factors [5], [6]. Previous studies have proposed that imbalances in hippocampal adult neurogenesis could be involved in the pathophysiology of depression and in the actions of antidepressant drugs, thus giving rise to a “neurogenic hypothesis” of depression [7]. Decreased proliferation and neuronal structural changes, within the hippocampus and other brain regions, are also increasingly recognized as key to the pathophysiology of depression [8].

To study stem cell activity *in vivo*, it is necessary to identify proliferating cells, but also to stably label their progeny so that other components of neurogenesis can be appreciated. Herein we describe a novel, non-invasive, method that combines BrdU immunohistochemistry with Golgi-impregnation; with this new method we can trace newborn neurons (BrdU labeling) and study their dendritic and spine structure, (3D morphometric analysis of Golgi-impregnated neurons) in distinct experimental conditions.

## Methods

### Ethics Statement

All procedures were carried out in accordance with National guidelines (Portaria n° 1005/92), with the European Union Directive 2010/63/EU and NIH guidelines on animal care and experimentation. This study was approved by the Ethical committee board of the Portuguese Veterinary Direction (DGV) as stated in the document with the reference 023248 in 11^th^ of October of 2005.

### Chronic mild stress protocol

Adult male Wistar rats (Charles-River Laboratories) were either handled (control) or submitted to a chronic mild stress (CMS) protocol [9]. To assure interference with neuronal proliferation, we administered methyazoxymethanol (MAM), an alkylating agent that arrests cellular proliferation (see **Methods S1**).

### Golgi-Cox staining

4 weeks after BrdU injections (100 mg/kg), rats were anaesthetized with sodium pentobarbital (Eutasil, 60 mg/Kg i.p.; Ceva Saúde Animal, Portugal) and perfused with 0.9% saline. Brains were removed, dropped into Golgi-Cox solution and kept in the dark for 15 days. Next, they were transferred to a 30% sucrose solution and kept in the refrigerator for 2 to 5 days in the dark until they sink. Sections (200 µm) were obtained in a vibratome (MicromHM-650V) and transferred to 24-well multiwell plate (Nunc) filled with distilled water for 15 min and then dipped in ammonium hydroxide (Sigma Aldrich) for 5 min in the dark. Sections were washed with distilled water twice, 10 min each, and dipped in Kodak Fix solution (Rapid fixer; Sigma Aldrich) for 20 min. After washes in distilled water, 10 min each, sections were dipped in PBS 1×, and kept cool in the refrigerator.

### BrdU Staining

After Golgi-Cox staining, sections were transferred to 6-well multiwell plates with citrate buffer (10 mM; pH = 6). For antigen retrieval sections were heated for 5 min in the microwave to near 100° to expose the BrdU epitope in the tissue, resulting in brighter fluorescent labeling of the cells that incorporated BrdU [10], [11] filter your current search. Sections were then rinsed in TBS 3 times, for 10 min and incubated with primary BrdU antibody (1∶50 in 0.5% Triton®-X 100 and 10% normal goat serum (NGS); rat anti-mouse, Novocastra) overnight at 4°C. The next day, sections were rinsed with TBS and incubated with secondary antibody (1∶1000 in 0.5% Triton®-X 100 and 10% NGS; anti-mouse Alexa Fluor 488, Invitrogen) for 2 h at RT. Finally, sections were incubated in DAPI (1 µg/ml) for 10 min at RT and then rinsed in TBS. Sections were mounted in superfrost slides (Menzel-Gläser) using Vectashield mounting medium (Vector Labs).

### Confocal microscopy and stereological analyses

Imaging of neurons was performed using an Olympus FV1000 laser scanning confocal microscope (emission wavelength 488 and brightfield) at high magnification (×40). Sections were optically sectioned using 1–2 µm intervals and cells rotated in orthogonal planes to verify double labeling. For each selected neuron that showed co-localization of Golgi-Cox with BrdU, all branches of the dendritic tree were reconstructed at 60× (oil) magnification using a motorized microscope (Axioplan2; Carl Zeiss) and Neurolucida software with the AutoNeuron extension module. A three-dimensional analysis of the reconstructed neurons was performed using NeuroExplorer software (Microbrightfield). For control rats, with and without MAM administration, 25 and 28 newborn neurons were reconstructed, respectively. For rats exposed to CMS, with and without MAM administration, 26 and 23 newborn neurons were reconstructed, respectively. For each neuron we examined the total dendritic length and the percentage of dendritic spine types. Three-dimensional Sholl analysis was used to evaluate the arrangement of the dendritic material. The analysis of spines was performed in segments visible for at least 30 µm in both proximal and distal branches of dentate granule cells. To assess changes in spine morphology, spines in the selected segments were classified into mushroom, thin, wide and ramified spines [8], [12] and the proportion of spines in each category was calculated for each neuron. In total, 30 branches were examined per experimental group. For control rats, with and without MAM administration, 420 and 215 dendritic spines were analyzed, respectively. For rats exposed to CMS, with and without MAM administration, 316 and 239 dendritic spines were analyzed, respectively.

### Statistical analyses

Two-way ANOVA was used to evaluate the dendritic arborisation and spine shape of newborn BrdU^+^ neuronal cells. Differences between groups were subsequently determined by Tukey's honestly significant difference test (Tukey HSD) post hoc analysis. Statistical significance was accepted for P<0.05. Results are expressed as mean±s.e.m.

## Results and Discussion

**Figure 1:**
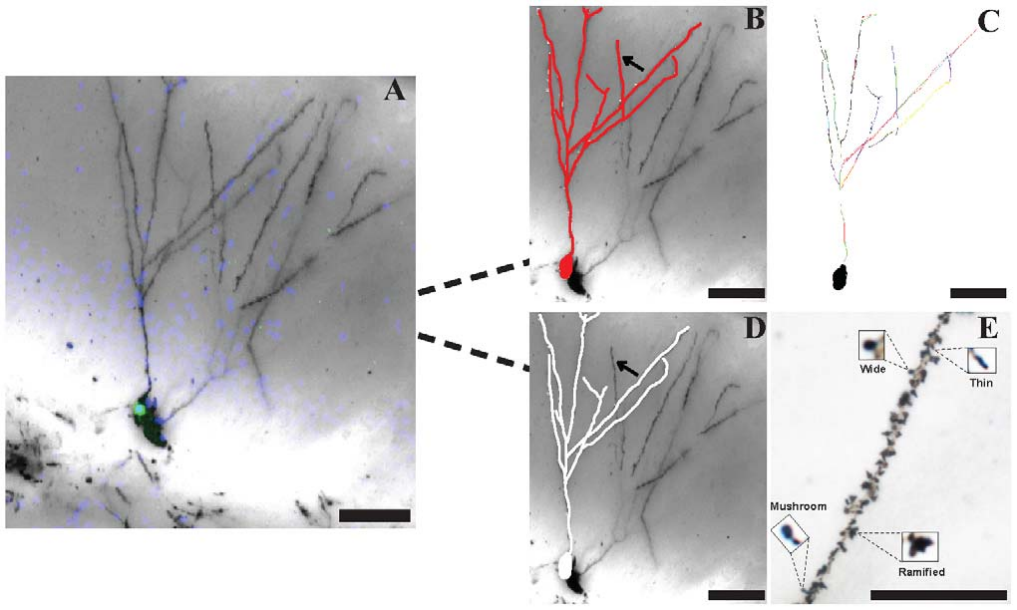
Three-dimensional analysis of a neuron double-labeled with BrdU and Golgi-Cox. Three-dimensional morphometric analysis of a Golgi-impregnated neuron co-labeled with BrdU (depicted with green dot) and Dapi (staining of nuclei depicted with blue dots) (**A**) using computer-assisted reconstructions. Neuronal reconstruction was performed using a motorized microscope and Neurolucida software with the automatic AutoNeuron extension module directly from the confocal image (red colour in **B** and **C**) and using manual reconstruction (white colour in **D**). Different colours on **C** depict distinct dendritic branches and black arrows in **B** and **D** depict the differences detected between the automatic and manual reconstructions. Image **E** depicts a neuronal segment showing all different spine types (mushroom, thin, wide and ramified). Scale bars: 50 µm.

**Figure 2:**
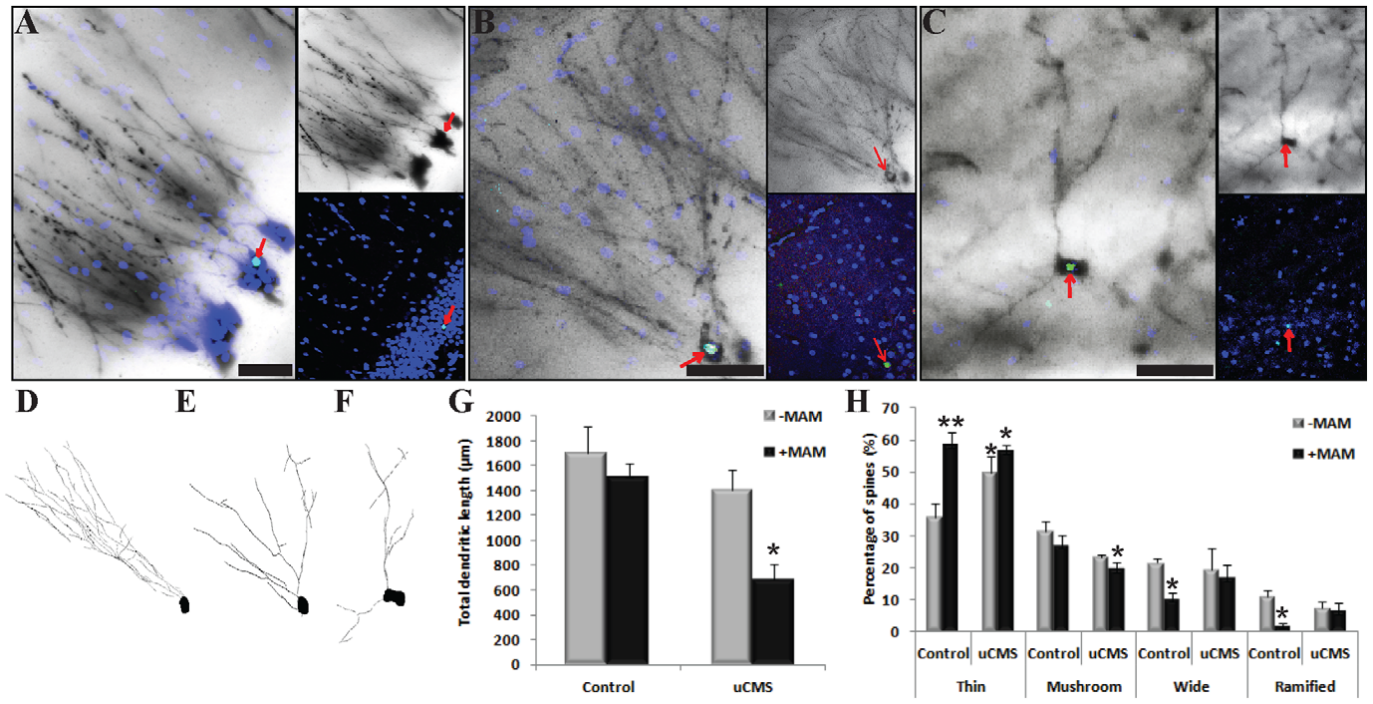
Confocal images and three-dimensional morphometric analysis of neurons double-labeled with BrdU and Golgi-Cox. (**A, B, C**) Confocal images of three dentate gyrus neurons double-labeled with BrdU (depicted with green dots) and Golgi-Cox (black staining). BrdU was administered for five consecutive days to 4 months old Wistar rats non-stressed and injected with saline for two weeks (Control) (**A**) or exposed to unpredictable chronic mild stress (uCMS) and injected with saline (**B**) or methyazoxymethanol (uCMS+MAM) (**C**) in the last two weeks of the stress protocol. Immunohistochemical analyses were performed 4 weeks after the injections. Nuclei (depicted with blue dots) were stained with Dapi. (**D, E, F**) Three-dimensional morphometric reconstruction analysis of the Golgi-impregnated dentate granule neurons double-labeled with BrdU shown in A (**D**) in B (**E**) and in C (**F**). (**G**) Graph showing the total dendritic length of newborn dentate granule neurons in the subgranular zone of different experimental groups (control and uCMS exposed rats untreated (−MAM) and treated with MAM (+MAM)). (**H**) Graph showing the percentage of different types of spines (thin, mushroom, wide and ramified) present in newborn dentate granule neurons in the subgranular zone of different experimental groups (control and uCMS exposed rats untreated (−MAM) and treated with MAM (+MAM)). Data represented as mean±se.m. Asterisk represents the comparison between control and all other experimental groups; *P<0.05 and **P<0.01. Scale bars: 50 µm.

We studied neurons that showed co-labeling of BrdU and Golgi-Cox staining using confocal microscopy (**Figure 1**, **2**) and performed three-dimensional morphometric analysis of the Golgi-impregnated neurons using computer-assisted reconstructions (**Figure 1**). To validate this method, we studied the structure of newborn neurons in the dentate gyrus (DG) of control rats (**Figure 1**, **2**) and compared to those of rats displaying depressive-like symptoms after exposure to CMS and/or to the administration of MAM, an alkylating agent that arrests cellular proliferation. All branches of the dendritic tree of the selected neurons were reconstructed using a motorized microscope and Neurolucida software with the AutoNeuron extension module (**Figure 1B, C**) and using manual reconstruction without the AutoNeuron extension (**Figure 1D**). No significant differences in total dendritic length per neuron were found between the automatic AutoNeuron and manual reconstructions, thus showing that distinct reconstruction strategies can be used to study the structure of these neurons. A three-dimensional analysis of the reconstructed neurons was performed using NeuroExplorer software (**Figure S1**). This method also allows the analysis of spine types (**Figure 1E**) which provides information on neuronal connectivity and synaptic plasticity [**13**].

The potential contribution of synaptic plasticity and neuronal connectivity to the development of, and recovery from, depressive-like behavior, has been scrutinized [8]. In these studies, the assessment of neurons dendritic arborization and spine shape was performed by three-dimensional morphometric analysis of Golgi-impregnated hippocampal neurons without making possible to distinguish between old and newly-born neurons. With this new method we could label neurons that were born during the CMS period using staining for BrdU and simultaneously study their cell shape, including dendritic processes. Thus, we could compare not only the morphology of newborn neurons in rats subjected to CMS, with and without MAM administration (**Figure 2B, C, E, F**), with those of control rats (**Figure 2A, D**), but also to compare them with adjacent pre-existent granule cells in the same animal. Here, we show that newborn neurons from the DG of rats subjected to CMS without blockage of neurogenesis (**Figure 2B, E, G**) are morphologically comparable to those of control untreated rats (**Figure 2A, D, G**). These findings also show for the first time that the newborn neurons from the DG of rats subjected to CMS and injected with MAM are unable to reach full dendritic development (**Figure 2C, F, G**). In contrast, control rats treated with MAM present normal dendritic architecture (**Figure 2G**).

Comparing the spine morphology of DG newborn neurons of control rats with those of rats exposed to CMS, with and without MAM administration, we found a significant increase in the percentage of thin spines in all animals treated with MAM (control and CMS) and in untreated CMS animals (**Figure 2H**). Moreover, CMS exposed rats present a significant reduction in the percentage of mushroom spines in newborn neurons when comparing to those of control rats. Newborn neurons of control and CMS exposed rats treated with MAM show a significant decrease in the percentage of thick and ramified spines in comparison to those of control untreated rats (**Figure 2H**). These alterations in the spine morphology of newborn neurons also suggest severe in the dendritic maturation of neurons in rats exposed to CMS and/or treated with MAM.

BrdU labeling is currently one of the prevailing methods to study proliferation and neurogenesis *in vivo*. Once injected systemically, BrdU is incorporated as a thymidine analog into the DNA of all cells undergoing DNA synthesis allowing its detection in postmitotic cells for the remainder of their life [14]. This technique was essential to the identification of the origin of newly-generated neuronal cells in the adult hippocampal SGZ [2], [15], [16]. So far, however, the analysis of the dendritic and synaptic structure of newly-born neurons could only be achieved by retroviral labeling [17], [18]. Retroviral labeling provides several advantages compared to BrdU labeling; for example, it allows distinguishing between cell division and DNA repair, as the stable integration of the retroviral genome into the chromosomal DNA can only happen after nuclear membrane breakdown. However, retroviral labeling has also several disadvantages that make it less suitable for *in vivo* studies comparing to BrdU labeling. Since the blood-brain barrier is an obstacle, retroviruses have to be applied directly into the region of interest through stereotaxic surgeries, causing brain lesions from the procedure and possible local inflammatory reactions. These lesions and inflammation may induce alterations on neurogenesis, thus raising some cautions when using this method to study stem cell activity in the brain. Moreover, this approach only permits the study of the region where the virus was injected and does not allow for precise temporal resolution of proliferation nor for the comparison of the neuronal morphology between old and newly-dividing cells.

Analysis of dendritic branching and spines by three-dimensional morphometric assessment of Golgi-impregnated neurons using computer-assisted reconstructions enables to generate a unique picture of the effect of different diseases and treatments on the fine neuronal structure. In fact, dendritic and synaptic pathology is a hallmark in several neuropsychiatric conditions. Standard histopathological techniques used to label neurons do not stain dendrites and spines and, thus, may miss aberrant dendritic branching and synaptic loss in neurodegenerative processes. In contrast, the Golgi-Cox staining is a simple and valuable method that provides detailed information on neuronal morphology allowing the detection of subtle damage. As shown herein, if combined with BrdU staining, this technique will further allow distinguishing whether these changes target specific neuronal populations (e.g. in the present study, whether they affect differently old versus newly-born neurons). Obviously, the applicability of this method is much broader, as the combination of other markers with Golgi staining will allow the analysis of the neuronal dendritic structure with its phenotypic characterization in a wide spectrum of experimental conditions. Indeed, a recent study shows the potential of combining Golgi-Cox staining with immunocytochemical staining, which permits the analysis of the morphological patterns of biochemically characterized neurons [19].

In summary, this novel simple and non-invasive method is an useful tool to study the fine neuronal structure in phenotypically characterized neurons in both physiological and pathological conditions. Its applicability is likely to be broad, if one considers all those conditions in which neurogenesis and dendritic/synaptic plasticity might be affected, such as in depression [8], Alzheimer's disease and schizophrenia [20].

## Supporting Information

Figure S1 **Three-dimensional analysis of a reconstructed neuron.** (**A–E**) Three-dimensional analyses of a reconstructed neuron using NeuroExplorer software. Panels **B–E** show the neuron from distinct 3D perspectives (coordinates).
(TIF)

Methods S1 **Chronic mild stress protocol.**
(PDF)
