# Supplementary figures and images for: Immuno-Golgi as a Tool for Analyzing Neuronal 3D-Dendritic Structure in Phenotypically Characterized Neurons
Source: PLoS One. 2012 Mar 12;7(3):e33114. doi: 10.1371/journal.pone.0033114 (PMC3299750; doi:10.1371/journal.pone.0033114)

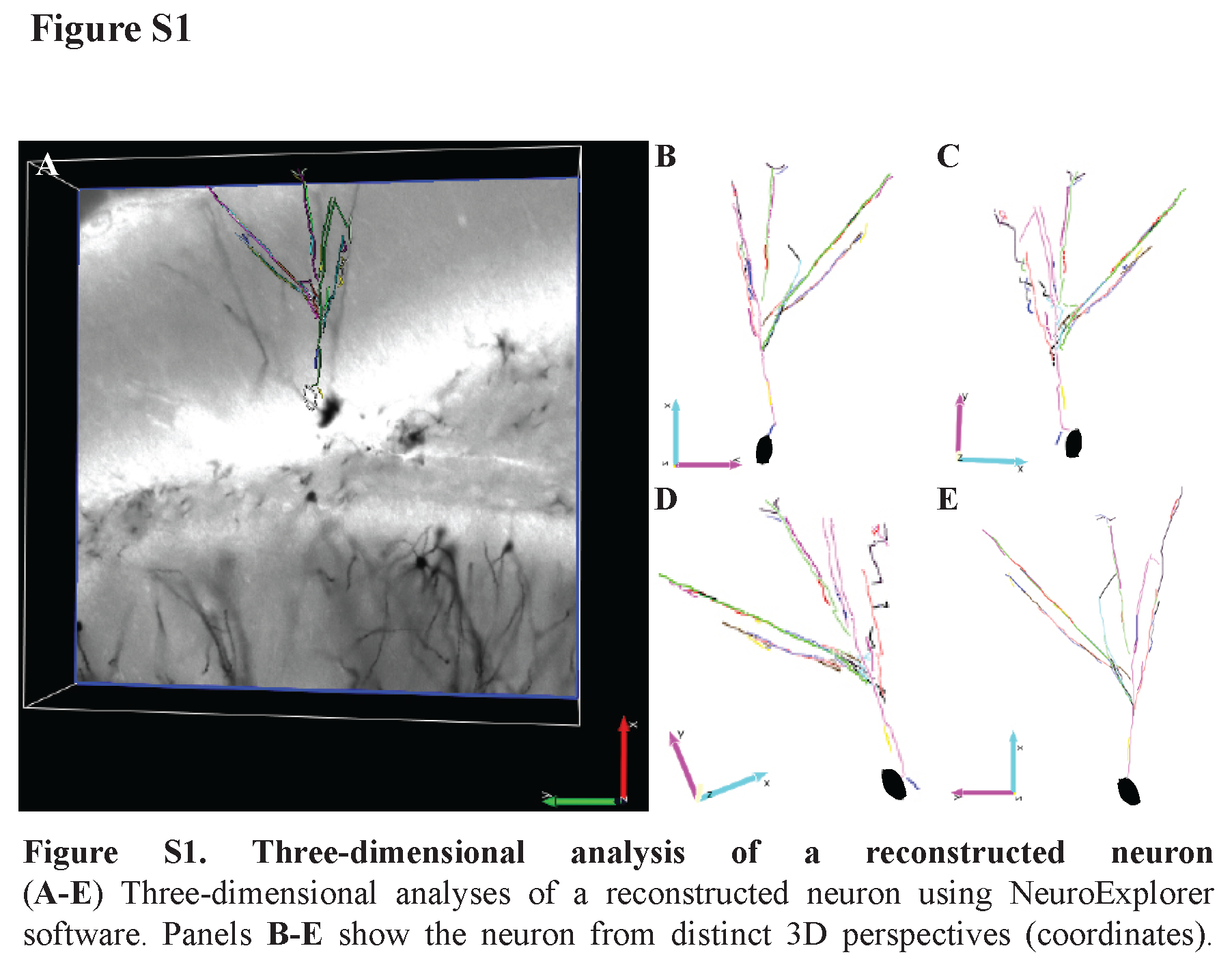

Supplement: Figure S1 — Three-dimensional analysis of a reconstructed neuron. (A–E) Three-dimensional analyses of a reconstructed neuron using NeuroExplorer software. Panels B–E show the neuron from distinct 3D perspectives (coordinates). (TIF) [file pone.0033114.s001.tif]
