# Supplementary material for: Immuno-Golgi as a Tool for Analyzing Neuronal 3D-Dendritic Structure in Phenotypically Characterized Neurons
Source: PLoS One. 2012 Mar 12;7(3):e33114. doi: 10.1371/journal.pone.0033114 (PMC3299750; doi:10.1371/journal.pone.0033114)
Supplement: Methods S1 — Chronic mild stress protocol. (PDF) [file pone.0033114.s002.pdf]

Methods S1

Chronic mild stress protocol

Male rats, weighing 200–300g and with 2 months of age were group-housed (three per cage) under 12h light: 12h dark cycles, at 22°C, relative humidity of 55% and with food and water *ad libitum*. Rats were distributed into 3 groups: 1 control group not exposed to uCMS and treated with saline; 2 groups exposed to uCMS and treated with either saline or MAM (7mg/Kg administered subcutaneously; National Cancer Institute, Midwest Research Institute, Kansas City, MO, USA) (n=14 rats per group). Briefly, the uCMS protocol encompassed several mild stressors (confinement to a restricted space for 1h; overnight food deprivation followed by 1h of exposure to inaccessible food; overnight water deprivation followed by 1h of exposure to an empty bottle; overnight damp bedding; inverted light/dark cycles; exposure to stroboscopic lights during 4h and noise exposure during 4h) to which animals were random- and uninterruptedly exposed during 6 weeks. In the following 4 weeks, the uCMS protocol was suspended and animals were allowed to recover, being kept unexposed to any kind of stressor. Administration of BrdU (100mg.kg-1 i.p.; Sigma Aldrich) was performed to all rats during 5 days with daily injections at the end of the stress period, encompassing the two last days of stress and the first three days of the recovery period.

All procedures were carried out in accordance with European Union Directive 2010/63/EU and NIH guidelines on animal care and experimentation.
